# Is One Sputum Specimen as Good as Two during Follow-Up Cultures for Monitoring Multi Drug Resistant Tuberculosis Patients in India?

**DOI:** 10.1371/journal.pone.0045554

**Published:** 2012-09-27

**Authors:** Sharath Burugina Nagaraja, Ajay M. V. Kumar, Kuldeep Singh Sachdeva, Ranjani Ramachandran, Srinath Satyanarayana, Avi Bansal, Malik Parmar, Sarabjit Chadha, Sreenivas Nair, Ashok Kumar, Sven Gudmund Hinderaker, Mary Edginton, Puneet K. Dewan

**Affiliations:** 1 Office of the WHO-Representative in India, World Health Organization, New Delhi, India; 2 Central TB Division, Ministry of Health and Family Welfare, New Delhi, India; 3 International Union Against Tuberculosis and Lung Diseases (The Union), South East Asia Region, New Delhi, India; 4 Centre for International Health, University of Bergen, Bergen, Norway; 5 International Union Against Tuberculosis and Lung Diseases (The Union), Paris, France; McGill University, Canada

## Abstract

**Background:**

In India, the Revised National Tuberculosis Control Programme (RNTCP) has adopted the strategy of examining two specimens during follow-up culture examinations to monitor the treatment response of multi-drug resistant tuberculosis (MDR-TB) patients.

**Objectives:**

To determine the incremental yield of the second sputum specimen during follow-up culture examinations among patients with MDR-TB and the effect on case management on changing from two to one specimen follow-up strategy.

**Methods:**

A cross sectional record review of MDR-TB patients registered during 2008–09 under RNTCP was undertaken in three MDR-TB treatment sites of India.

**Results:**

Of 1721 pairs of follow-up sputum culture examinations done among 220 MDR-TB patients, 451(26%) were positive with either of the two specimens; 29(1.7%) were culture positive only on the second specimen indicating the incremental yield. To detect one additional culture positive result on the second specimen, 59 specimens needed to be processed. If we had examined only one specimen, we would have missed 29 culture-positive results. By current RNTCP guidelines, however, a single specimen policy would have altered case management in only 3(0.2%) instances, where patients would have missed a one month extension of the intensive phase of MDR-TB treatment. There is no meaningful advantage in using two specimens for the monitoring of MDR-TB patients. A single specimen policy could be safely implemented with negligible clinical effect on MDR-TB patients and favourable resource implications for RNTCP.

## Introduction

In India, the estimated incidence of multi-drug resistant (MDR) tuberculosis (TB) cases in 2009 was 99,000. [1] The Revised National TB Control Programme (RNTCP) began implementing the programmatic management of drug resistant tuberculosis (PMDT) in a phased manner from 2007, and is rapidly scaling up PMDT services. By the end of 2011, RNTCP had initiated 6994 patients on MDR-TB treatment [2].

In accordance with international and national guidelines, the progress of each patient during MDR-TB treatment is monitored by sputum culture at a Culture and Drug susceptibility testing (C&DST) laboratory accredited by the national programme. The purpose of follow-up culture monitoring is to assess the response to MDR-TB therapy, in order to guide treatment adjustments. A positive culture indicates poor response to therapy; conversely consistently negative cultures indicate good response. Although collection of two sputum specimens to maximize culture recovery has been recommended for the diagnosis of MDR-TB cases and in international guidelines for the surveillance of drug-resistant tuberculosis, international guidelines are silent as to the number of sputum specimens recommended for follow-up culture examinations in MDR-TB [3]–[4].

RNTCP in national PMDT guidelines has chosen to collect two specimens for both diagnosis of MDR-TB and for follow-up culture monitoring. The schedule for monitoring involves examining two sputum specimens at months 3, 4, 5, 6, 7, 9, 12, 15, 18, 21, 24 (11 monitoring follow-ups) (Table 1) [5]. The first and second specimens are recorded as ‘A’ and ‘B’ specimens respectively in the C&DST register. The rationale for performing two specimens for sputum culture at each follow-up visit is to have high sensitivity for the detection of positive cases which would have an impact both programmatically and on case management of multi-drug resistant TB patients especially in the intensive phase. The disadvantage is that large numbers of specimens have to be examined in restricted laboratory capacity settings, with unknown additional impact of second specimen on patient management. With the current follow-up schedule and policy of examining two specimens during every follow-up of an MDR-TB patient, and given the rapid expansion of PMDT services in the country, the RNTCP has estimated approximately 880,000 thousand follow-up sputum culture examinations will be required, each requiring two sputum specimens [2].

**Table 1:** The Existing Schedule for sputum smear microscopy, culture and sensitivity follow-up examinations in RNTCP.

Schedule for sputum smear microscopy, culture and sensitivity follow-up examinations														
	Intensive phase (IP) monthly follow-up examinations				Extension of IP (1–3 months)			Continuation Phase (CP) Quarterly follow-up examination in months						
	1^st^ FU	2^nd^ FU	3^rd^ FU	4^th^ FU				I qtr		II qtr	III qtr	IV qtr	V qtr	VI Qtr
No IP extension→	3	4	5	6	–	–	–	7	9	12	15	18	21	24
IP extension 1 month →	3	4	5	6	7	–	–	8	10	13	16	19	22	25
IP extension 2 months→	3	4	5	6	7	8	–	9	11	14	17	20	23	26
IP extension 3 months→	3	4	5	6	7	8	9	10	12	15	18	21	24	27

A single-specimen policy for follow-up culture examinations would be expected to reduce patient inconvenience, the burden of specimens requiring processing in laboratories and costs. This in turn would mean more available bandwidth for the processing of diagnostic specimens for the detection of MDR-TB. Despite these broad ramifications, there was no information to guide this policy decision. We conducted a retrospective study at three MDR-TB treatment sites in India to determine the incremental yield of a second sputum specimen for culture during follow-up examinations of MDR-TB patients registered during 2008–09 and to determine the impact of changing from two to a single specimen follow-up strategy on case management.

**Table 2:** Incremental yield of second specimen during follow-up culture examinations among Multi Drug Resistant Tuberculosis patients registered in 2008–09, India.

Total	1721
**Culture results of first and second specimen**	
Negative-Negative	1226
Negative-Positive	18
Negative-Contaminated	4
Positive-Negative	28
Positive-Positive	390
Positive-Contaminated	4
Contaminated-Negative	8
Contaminated-Positive	11
Contaminated-Contaminated	32
**Essential patterns of culture examination results**	
Positive-Any result	422
Non positive-Positive	29
Rest	1270
**Number (Proportion) positive on any specimen**	451 (26.2%)
**Number (Proportion) positive on second specimen only: Incremental Yield**	29 (1.7%)
95% Confidence Interval	1.0%–2.4%
**Additional number of culture examinations to detect one positive result on second specimen**	59
95% Confidence Interval	42–102

## Methods

### Design

This is a cross sectional study involving review of programme records and registers.

**Table 3:** Impact of examining only one specimen instead of two for culture during follow-up of multi-drug resistant tuberculosis patients, India, 2008–09.

Patient ID	Results of monthly serial culture examinations during thecourse of MDR-TB treatment*	Final treatment outcome	Clinical impact
1-2-002	–01110***1***–-1–1–-1-1––-1–	Failure	No
1-2-007	–***1***1111111–––––––––-	Death	No
1-2-008	–00000-1–1–111***1***–0–1––-	Failure	No
1-2-034	–0100100––-0–***1***–1––––	Death	No
1-2-040	–-10000-1–1–1–***1***–––––	Death	No
1-2-049	–-111111––-1–***1***–1–1–***1***–	Failure	No
1-2-056	–0000***1***-0–0–0–0–0–0––-	Cured	No
1-2-061	–***1***0-0–1–0–0––-0–0––-	Cured	No
1-2-065	–1111–***1***-1–-1–1–1–1––-	Failure	No
1-2-066	–***1***00–-0–0–0-1–-1–1––-	Failure	No
2-1-009	–1111–***1***–1–0–1–1–***1***–***1***–	Failure	No
2-2-001	–***1***1100000-0–0–0–0–0–0–	Cured	No
2-2-008	–10***1***1––––***1***–––––––	Death	Yes**
2-2-010	–1110100-1–1–0–***1***––––-	Treatment completed	No
2-2-015	–111***1***1110-1–0–1–––––-	Death	No
2-2-016	–1000–0–***1***–1–***1***–1–1––-	Failure	No
2-2-022	–11101-1–***1***–1–1––-1–1–	Failure	No
2-2-023	–***1***1***1***000***1***01–1–1–1–1–***1***–-	Failure	Yes**
2-2-024	–1111***1***1***1***–1–1–1–––0––	Treatment completed	No
2-2-026	–111***1***-100-0–0–-0-0–0–0–	Cured	Yes**

* These are the results of follow-up culture examinations starting from month 1 to month 29 as applicable. 1 indicates culture positive result, 0 indicates culture negative result and “–” indicates either follow-up not done or not applicable; ***1*** indicates positive culture results which would have been missed if only one specimen had been examined.

** Would have missed extending Intensive phase of treatment by one month.

### Setting

Under India’s RNTCP, the diagnosis of MDR-TB is made by doing Culture and Drug Susceptibility Testing (C&DST) on patients who fail RNTCP treatment regimens. The diagnosed MDR-TB patients undergo pre-treatment evaluation before starting MDR-TB treatment at MDR-TB treatment sites where the patient is registered, started on treatment and monitored in hospital for a period of at least seven days before referral to domiciliary treatment.

Treatment is given in two phases, Intensive (IP) and continuation (CP). The total duration of treatment for regimen of MDR-TB is 24–27 months, depending on the IP duration which should be given for at least six months. Patients will be considered culture converted after having two consecutive negative cultures taken at least one month apart in IP. After six months of treatment, patients are reviewed and treatment changed to CP if both the 4^th^ and 5^th^ month culture results are negative. If either or both 4^th^–5^th^ month culture results remain positive, treatment is extended by one month. Extension of IP beyond 1 month will be decided on the results of sputum culture of 5^th^ or 6^th^ and 6^th^ or 7^th^ months. If the 4^th^ month culture is still awaited after 6 months of treatment, the IP is extended until the result is available, with further treatment being decided according to the culture results. The IP can be extended up to a maximum of 3 months after which the patient will be initiated on CP irrespective of the culture result. The recommended duration of CP is 18 months.

For follow-up examinations, the specimens are collected and transported under cold chain conditions from the respective District TB Centre and examined by smear and culture at least 30 days apart from the 3^rd^ to 7^th^ month of treatment (i.e., at the end of the months 3,4,5,6 and 7) and at 3 monthly intervals from the 9^th^ month onwards till the completion of treatment (i.e., at the end of the months 9, 12, 15, 18, 21 and 24). All the specimens were subjected to solid culture. During the study period, the C & DST laboratories of these study sites were accredited by RNTCP for solid culture technology. The WHO standardised treatment outcome definitions are used in RNTCP.

All MDR-TB patients registered at three MDR-TB treatment sites during 2008–09, and whose treatment outcome were reported by end of 2011 were included in the study. The three sites were Hyderabad (Andhra Pradesh), Ahmedabad (Gujarat) and Jaipur (Rajasthan).

### Variables and Data Collection

Variables collected for each patient were patient name, DOTS-plus TB number, age, sex, HIV status, date of registration, month of follow-up, date and results of smear examination, date and results of culture examination and treatment outcome. The outcome variables were all culture results. Data were collected into a structured proforma by the trained staff at MDR-TB treatment sites and laboratories. The source of data was from DOTS-plus TB register and C&DST laboratory register.

### Analysis and Statistics

All the data was entered twice by independent data entry operators trained for the purpose into an EpiData database. The two databases were then compared for discrepancies and a final database was created after correcting discrepancies by referring the original records. This final database was then analyzed using EpiData analysis software. Data were cross-tabulated and the following were calculated: the proportion of follow-up examinations which were culture positive, the proportion who were positive on the second specimen only when the first specimen was non positive (i.e., the “incremental yield” - X), and the its inverse (1/X) was determined to calculate the number of second sputum culture examinations, that were done to get one additional culture positive case.

The longitudinal culture results of the MDR-TB patients were then examined to estimate hypothetically, the impact of using the results of single (first) specimen only on case management of MDR-TB patients; specifically to find the number of patients who would have missed IP extension, number who would have delayed submission of specimens for second line drug susceptibility testing and number who would have a different outcome declared or led to a delay in declaration of outcome.

### Ethics Approval

As this study was a record review of routinely collected RNTCP data, informed consent of individual patients was deemed unnecessary. The same was reviewed and approved by Central TB Division, Ministry of Health and Family Welfare, India andthe Ethics Advisory Group of International Union Against TB and Lung Disease (The Union).

## Results

A total of 220 MDR-TB patients registered at the three MDR-TB treatment sites were included in the study. During their treatment period 1763 pairs of follow-up culture examinations were done. Data of 19 first culture examination results and 23 second culture examination results were not recorded and hence results of remaining 1721 (98%) pairs were included in the analysis.

### Incremental Yield of the Second Sputum Specimen for Culture during Follow-up Visits

Of 1721 pairs of follow-up sputum culture examinations done among 220 MDR-TB patients, 451(26%) were positive with either of the two specimens; 29(1.7%) were culture positive only on the second specimen indicating the incremental yield. (Table 2) To detect one additional culture positive result on the second specimen, 59(42–102) specimens needed to be processed.

### The Effect on Case Management on Changing from Two to Single Specimen Follow-up Strategy

As shown in Table 1, of 1721 follow-up examinations, there were a total of 29 (1.7%) instances where we would have missed a positive culture result had we adopted a single specimen strategy. The results of serial follow-up culture results of MDR-TB patients in whom we would have missed a positive culture had we performed culture examination of only one specimen and the possible impact on case management is shown in Table 3. In only 3 instances (0.2% of all follow-up examinations), the missed positive cultures would have impacted on case management in terms of failing to extend the intensive phase by one month. There was no impact of missed positive cultures on declaration of treatment outcome or initiating action for assessing second line drug susceptibility testing.

## Discussion

This is the first study describing the incremental yield of the second sputum specimen during follow-up culture examinations of MDR-TB patients and the impact on case management on changing from two to single specimen follow-up strategy. The strength of the study is that routine programme data was used from the cohort of MDR-TB patients registered under RNTCP, thus reflecting the operational reality. We have adhered to STROBE guidelines on reporting [6].

These findings have the following programmatic implications. First, the incremental yield of examining a second specimen was very low at 1.7%, and it is pragmatically redundant to process nearly 60 follow-up specimens to get one additional positive culture. In resource limited settings with limited laboratory capacity, reducing to single specimen follow-up will decrease the laboratory workload considerably and helps in prioritizing the effort towards examining more diagnostic specimens [7]. This will help in diagnosing more MDR-TB patients who are in need to treatment as currently only 3–4% of all estimated MDR-TB cases in the country are on treatment. In addition, there would be reduced direct and indirect logistics cost involved in sputum transportation and processing. Patient treatment adherence may improve if they are relieved of the burden of extra visits to health facilities to provide sputum specimens. This we believe is a crucial step in advancing towards realising the ambitious plan of RNTCP to achieve universal access to diagnosis and treatment of all MDR-TB patients in the country.

### What is the Effect of Single Specimen Follow-up Strategy on Clinical Case Management?

The effect of the single specimen follow-up strategy was limited to a handful of missed positive cultures which in turn would have had very little effect on MDR-TB case management. Effectively, the collection transportation and laboratory processing of 1721 second follow-up cultures led to 3 instances where the intensive phase of treatment is extended. Reassuringly, there were no observed lost opportunities for the detection of treatment failure and referral for second line drug susceptibility testing. Rather, any possible events would have been triggered by an earlier or a later follow-up culture examination. With serial cultures, such events would be likely caught at the next or preceeding follow-up cultures. This is very convincing evidence to move towards single specimen follow-up culture examination and urge RNTCP to consider policy change in this regard.

Though, we did not find any instance in this cohort of MDR-TB patients, the other possible impact on case management of a missed positive culture is that we miss the opportunity to identify treatment failure early and initiate steps to examine if the patient has extensively drug resistant tuberculosis. This possibility can be greatly minimized if national programme takes a policy decision to examine all the specimens for second line DST right at the beginning of treatment. However, the current capacity of RNTCP for second line drug susceptibility testing is limited and needs to be strengthened further.

This study had some limitations. As in any record–based study, it is limited by the retrospective nature of the data, which may have been inaccurate or incomplete; however, routine supervision and monitoring within the programme is likely to have reduced this. [8] The study is also limited by the fact that the first follow-up specimen sent for C&DST may either be spot or early morning sputum specimen as the time of sputum collection was not documented by health staff.

In conclusion, there is no meaningful advantage in using two specimens for the monitoring of MDR-TB patients. A single specimen policy could be safely implemented with negligible clinical effect on MDR-TB patients and favourable resource implications for RNTCP.
